# Retinochoroidal toxoplasmosis in a patient with cerebral post-transplant lymphoproliferative disease of Hodgkin’s type: a diagnostic challenge

**DOI:** 10.1186/s12348-015-0055-y

**Published:** 2015-08-04

**Authors:** Ruchi Mittal, Gabrielle Thumann, George Souteyrand, David Kuerten, Sarah E. Coupland

**Affiliations:** Dalmia Ophthalmic Pathology Services, L.V. Prasad Eye Institute, Bhubaneswar, India; Département des neurosciences cliniques, Service d’ophthalmologie Unité du segment posterieur, Hôpitaux Universitaires de Genève, Geneve, Switzerland; Department of Ophthalmology, RWTH Aachen University, Aachen, Germany; Liverpool Ocular Oncology Research Group, Department of Molecular and Clinical Cancer Medicine, Institute of Translational Medicine, University of Liverpool, 6th Floor Duncan Building, Daulby Street, Liverpool, L69 3GA UK

**Keywords:** Retinochoroidal toxoplasmosis, Renal transplant, Post-transplant lymphoproliferative disease, Retinal necrosis, Toxoplasmosis infection, Hodgkin’s disease, Uveitis, Oculocerebral, Inflammation, Histology

## Abstract

Toxoplasmosis is a relatively rare complication in renal transplant patients and can pose diagnostic challenges, especially when it manifests as an ocular inflammation. Authors hereby report an unusual case of a 57-year-old male who developed retinochoroidal toxoplasmosis after 15 years of renal transplant, the diagnoses of which were challenging as the patient was also a known case of cerebral post-transplant lymphoproliferative disease (PTLD) of Hodgkin’s type, which misled the ophthalmologists towards a clinical diagnosis of ocular PTLD. Histopathology examination of the enucleated eye revealed numerous toxoplasmosis cysts within the retina and choroid. No ocular PTLD was observed.

## Correspondence

Although reported previously, retinochoroiditis due to toxoplasmosis is relatively rare in renal transplant patients [1]. A definitive timely diagnosis in such cases can be challenging, particularly in atypical clinical presentations, as in our patient, who was also diagnosed with cerebral post-transplant lymphoproliferative disease (PTLD) of Hodgkin’s type [2, 3]. The presentation of bilateral extensive uveitis due to retinochoroidal toxoplasmosis 15 years after renal transplantation, but clinically suspected to be an oculocerebral PTLD, has not been reported previously to our knowledge.

## Case description

**Fig. 1:**
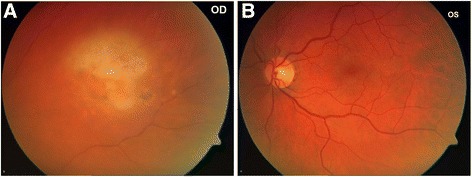
Colour fundus photographs of the right **a** and left **b** eyes, taken in 2006

A 57-year-old male presented to Aachen University Eye Clinic in June 2009 with bilateral uveitis and vitritis. He had undergone a renal transplant for chronic glomerulopathy in 1990 and had received immunosuppression with cyclosporin A, mycophenolate mofetil and fortecortin. He suffered episodic uveitis in the left (OS) and right (OD) eyes in 2003 and 2006, respectively (Fig. 1). On both occasions, vitreous biopsies did not reveal definitive infectious causes (e.g. viral or protozoa) in either real time-polymerase chain reaction (RT-PCR) analyses or in bacterial and fungal cultures. Furthermore, atypical cells were not observed within the reactive lymphocytic infiltrates on microscopy. Serological examinations ruled out an infectious aetiology at this stage. On both occasions, vancomycin was administered intraocularly, whilst cefuroxime was given systemically. This led to a resolution of the ocular inflammation and ultimate development of focal retinochoroidal scars without evidence of hyperpigmentation in both eyes.

**Fig. 2:**
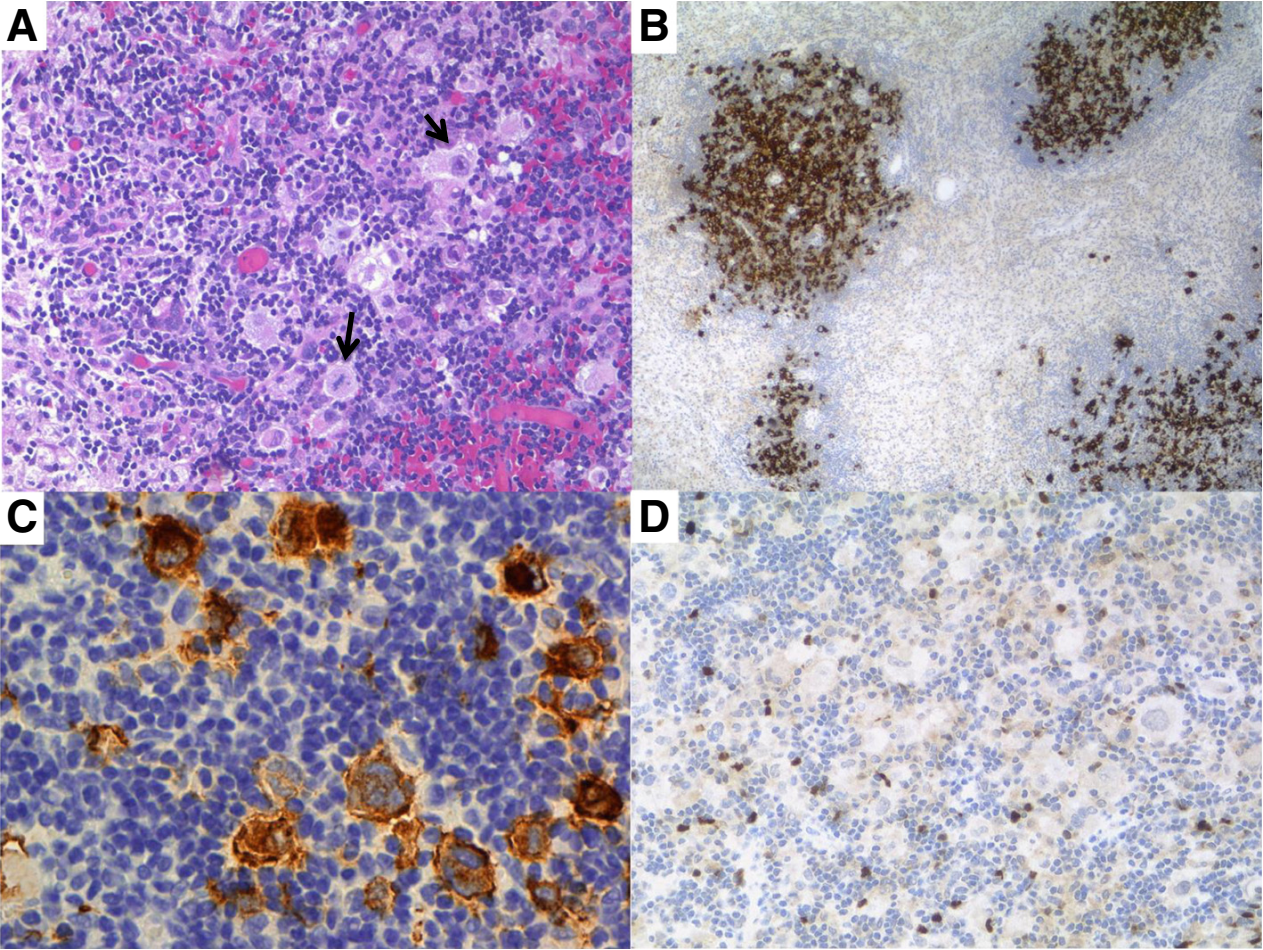
**a** Photomicrograph of the cerebral biopsy showing numerous mononuclear Hodgkin cells and multinucleated Reed Sternberg (HRS) cells in a cellular background (*arrows*) (haematoxylin-eosin; original magnification ×200). HRS cells are positive for **b** CD30 (PAP immunostaining; original magnification ×100) and strongly express the **c** Epstein Barr virus-encoded latent membrane protein-1. **d** BOB.1 is negative in the HRS cells and is positive in bystander cells (PAP immunostaining; original magnification ×400) (Images courtesy of Prof. I. Anagnostopoulos)

In 2007, the patient developed right-sided leg weakness and paralysis. Computerised tomographic (CT) scans revealed a left frontal lobe lesion. A stereotactic biopsy was performed, and a rare cerebral PTLD of Hodgkin’s type was diagnosed, positive for CD30 and Epstein Barr virus (EBV)-encoded latent membrane protein-1 (Fig. 2a–d). Importantly, there was no histological evidence of dual pathology—i.e. there was no evidence of microorganisms. The patient was treated with external beam radiotherapy (40 Gy) in divided doses.

**Fig. 3:**
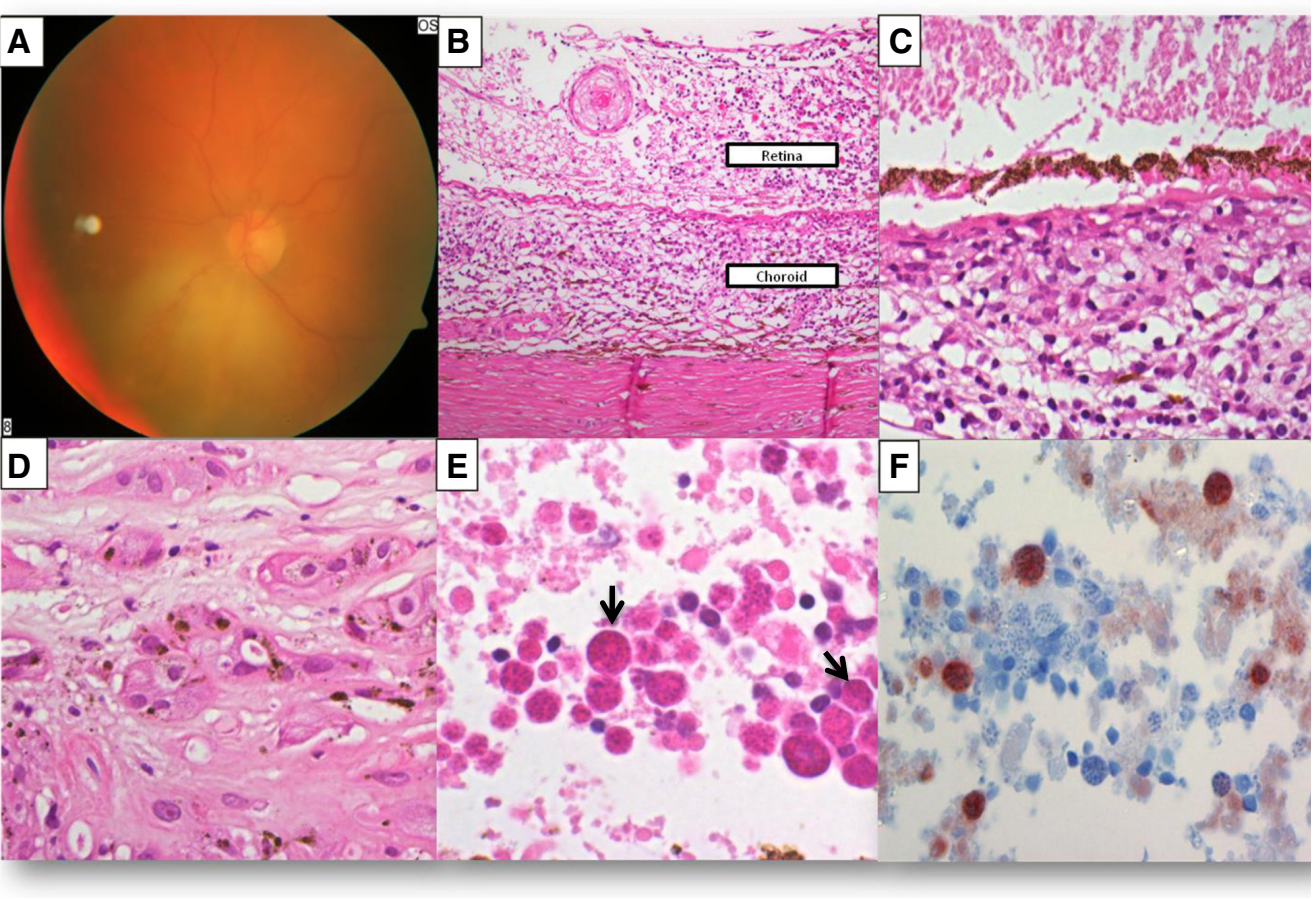
**a** Colour fundus photograph of the left eye shows extensive yellowish white retinochoroidal infiltrates. **b** Photomicrograph shows chronic inflammation of the choroid and retina (haematoxylin-eosin; original magnification ×100), with **c** retinal necrosis (haematoxylin-eosin; original magnification ×200). **d** Scarring of the retina with hyperplasia of retinal pigment epithelium was noted focally (haematoxylin-eosin; original magnification ×400). **e** Necrotic retina shows numerous cysts of toxoplasma (*arrows*) (haematoxylin-eosin; original magnification ×1000). **f** Cysts are positive for antibodies to *Toxoplasma gondii* (PAP immunostaining; ×1000)

In March 2009, the patient developed a rhegmatogenous retinal detachment OD, arising in the area of the residual retinochoroidal scars, which did not show any signs of reactivation; a vitreous biopsy performed at that time did not reveal any further information. On examination in June 2009, the patient had light perception only OD with a raised intraocular pressure (IOP) of 23 mmHg and a visual acuity of 0.1 OS. On fundoscopy, there were numerous extensive yellowish white retinochoroidal infiltrates OS, extending to the peripapillary region (Fig. 3a). In view of the previous history of cerebral PTLD, a clinical diagnosis of presumed oculocerebral PTLD was made, and the treatment option with intravitreal methotrexate was considered; however, confirmation of clinical diagnoses was required. Since the OD was nearly blind and painful with a raised IOP of 23 mmHg, and considering that the previous vitreous biopsy was non-informative, the right eye was enucleated.

Histological sections demonstrated an enlarged globe with focal scleral thinning. Moderately dense chronic inflammatory infiltrates were seen in the anterior chamber, iris stroma, ciliary muscles, anterior vitreous and entire choroid, with focal vasculitis (Fig. 3b). The retina was near-to-completely necrotic (Fig. 3c, asterisk marked) with the remaining viable areas being detached. Focal areas of retinal scarring, with RPE proliferation at the scar edges, were seen (Fig. 3d). Within the necrotic retina, numerous toxoplasmosis cysts were observed (Fig. 3e, arrows); these were confirmed on immunohistochemistry (Fig. 3f). There was no evidence of any Hodgkin-like CD30+/EBV+ cells despite numerous levels being performed. A diagnosis of extensive chronic uveitis with necrotic retinochoroiditis caused by *Toxoplasma gondii* was made. Subsequent cerebrospinal fluid and serum examinations were positive for *T. gondii* by PCR and enzyme-linked immunosorbent assay. This contrasted subsequent cerebral biopsies, which showed neither toxoplasmosis cysts nor residual PTLD cells. Furthermore, the blood toxoplasma IgG levels were only slightly elevated, and IgM was negative. The patient received treatment with combination of pyrimethamine, sulfadiazine and clindamycin; however, he unfortunately succumbed to the combined disease processes shortly thereafter.

## Discussion

This case illustrates a number of unusual pathologies and clinical challenges. Firstly, it describes a case of cerebral PTLD of Hodgkin-type, which has been never reported in the literature. Secondly, it exemplifies the fact that an open mind must be kept in the differential diagnosis of immunosuppressed patients with a “masquerade syndrome”-like presentation. Although the main clinical suspicion in the present case was intraocular involvement of the cerebral PTLD, the differential diagnosis of intraocular toxoplasmosis was equally highly ranked, considering the medical history of the patient. Fulminant toxoplasmosis in post-transplant patients may occur as a primary infection shortly after transplantation or as reactivation of latent infection, even up to 7 years after transplantation [4]. It may have variable uveal involvement and can progress rapidly to panophthalmitis and even orbital cellulitis [5]. Hence, dual pathologies coexisting in a single patient, possibly at the same [6] or different sites, must always be considered, and early diagnosis of these entities, although challenging, is essential.

## Abbreviations

EBV: Epstein Barr virus
IOP: intraocular pressure
OD: right
OS: left
PTLD: post-transplant lymphoproliferative disease
